# Impact of Glycosylated Fish Gelatin Emulsion Gels on the Gel Properties and Structural Characteristics of Surimi Gels

**DOI:** 10.3390/foods15081434

**Published:** 2026-04-20

**Authors:** Huaiyuan Chen, Jiaqi Huang, Xinxin Fan, Ru Jia, Changrong Ou, Huamao Wei, Tao Huang

**Affiliations:** Zhejiang–Malaysia Joint Research Laboratory for Agricultural Product Processing and Nutrition, College of Food Science and Engineering, Ningbo University, Ningbo 315211, China; 19858992933@163.com (H.C.); 19560511659@163.com (J.H.); 15888289813@163.com (X.F.); jiaru@nbu.edu.cn (R.J.); ouchangrong@nbu.edu.cn (C.O.); weihuamao@nbu.edu.cn (H.W.)

**Keywords:** surimi, glycosylated fish gelatin, emulsion gels, gel properties, lipid enrichment, texture, moisture distribution

## Abstract

Surimi-based products are widely popular in the market owing to their unique texture and nutritional properties; however, traditional processing methods often result in reduced lipid content, despite lipids playing a crucial role in health. This study evaluated the effects of adding glycosylated fish gelatin emulsifying gel (prepared by glycosylating fish gelatin (FG) with D(+)-glucose (Glu) or β-cyclodextrin (β-CD) for 2 h) at 5%, 10%, and 15% (*w*/*w*) to hairtail surimi on its gel properties. The results indicated that both emulsified gels significantly enhanced gel strength, texture, and visual whiteness of hairtail surimi gel, with FG-βCD showing more pronounced improvements. FG-βCD also substantially reduced exudation and improved moisture distribution, resulting in a 69.81% decrease in juice loss. Furthermore, the addition of gelatin emulsifying gels shifted protein secondary structures toward more ordered forms, increasing α-helix and β-sheet content while reducing disordered components. Chemical interaction analysis revealed that hydrophobic interactions and nonspecific binding contributed to the reinforcement of gel formation. In conclusion, these findings highlighted that glycosylated emulsifying gels, as functional exogenous additives for surimi, offer a viable strategy for developing lipid-enriched, high-quality surimi products that meet emerging nutritional demands.

## 1. Introduction

Ribbonfish surimi is an important raw material in the aquatic processing industry, valued for its high-quality protein and nutrient-rich composition, which underpin its use in a variety of seafood products [1]. Nevertheless, during processing, conventional ribbonfish surimi frequently exhibits limitations in gel strength, darker color, and insufficient water-holding capacity [2]. These drawbacks considerably constrain its market appeal and consumer acceptance. Hence, improving the gelling performance of ribbonfish surimi remains a key focus for researchers and manufacturers in the field of aquatic foods.

Recently, emulsion gels have emerged as a promising strategy for modifying surimi-based products [3]. Studies have indicated that emulsion gels can significantly enhance the stability and strength of surimi gels by reinforcing interactions among surimi proteins [4]. This improvement is likely attributable to the formation of a robust gel network, which effectively improves water-holding capacity and reduces moisture loss during processing. Furthermore, the emulsifying properties of these gels increase their hydrophilicity [5], promoting stronger binding between water and surimi proteins and ultimately leading to a more uniform and resilient gel structure. Numerous studies have demonstrated that glycosylation can improve the properties of protein emulsions; however, reports on the effects of glycosylated protein emulsion gels on fish paste gel quality remain scarce.

Fish gelatin, as a safe natural ingredient, can be used in food as a gelling agent and emulsifier. However, fish gelatin exhibits poor emulsifying properties, which limit its commercial application values. Research indicates that glycosylation modifications can enhance its emulsifying and gelling capabilities, with its performance significantly influenced by glycosylation factors (e.g., glycoprotein ratio, reaction time, temperature, and pH). Through glycosylation modification using glycosylating agents such as D-galactose, methoxylated pectin, or k-carrageenan, functional properties can be effectively regulated to enhance emulsification and gelling performance [6]. Previous studies have demonstrated that several factors during the glycosylation process—including the sugar-to-protein ratio, reaction time, temperature, and pH—significantly affect gel properties [7,8]. This formed emulsion-gel system by glycoconjugate-modified proteins combines the encapsulation properties of emulsions with the three-dimensional network structure of gels. It offers an innovative raw material for developing fat-free, high-protein foods and holds broad application prospects in the food industry. However, the application of glycosylated gelatin emulsion gels has been less reported.

Though some reports have established the efficacy of glycosylation in enhancing the functional properties of protein emulsions, the application of glycosylated protein emulsion gels within surimi systems remains limited. A critical research gap exists in understanding how the molecular configuration of glycosylating agents—specifically linear monosaccharides versus cyclic oligosaccharides—modulates the functional performance of surmi gels as additives. Consequently, this study systematically investigates the influence of D(+)-glucose (Glu) or β-cyclodextrin (β-CD) glycosylated fish gelatin (FG) emulsion gels on the gelling characteristics of hairtail surimi. By evaluating these structurally distinct modifiers, we aim to elucidate the mechanisms by which glycosylated carriers modulate protein-lipid-water interactions, thereby providing a novel technical framework for the development of high-quality, lipid-enriched surimi products.

## 2. Materials and Methods

### 2.1. Materials and Reagents

Ribbon fish surimi was obtained from Ningbo Lanyang Aquatic Products Co., Ltd. (Ningbo, China); and the BCA Protein Concentration Assay Kit was obtained from Beijing Solarbio Technology Co., Ltd. (Beijing, China). Fish gelatin (FG, Type B) was sourced from Shanghai Yuan Ye Biotechnology Co., Ltd. (Shanghai, China); and o-phthalaldehyde (OPA) and dithiothreitol (DTT) were obtained from Shanghai Aladdin Biochemical Technology Co., Ltd. (Shanghai, China); D(+)-glucose (Glu), hydrochloric acid, sodium hydroxide, borax, and sodium dodecyl sulfate (SDS) provided by Sinopharm Group Chemical Reagent Co., Ltd. (Shanghai, China); β-cyclodextrin (β-CD) from Beijing Solabao Technology Co., Ltd. (Beijing, China); and soybean oil from Yihai Kerry Golden Dragon Fish Grain & Oil Food Co., Ltd. (Shanghai, China). And other reagents were of analytical grade.

### 2.2. Preparation of Surimi Gel

Preparation of Glycosylated Fish Gelatin Emulsions: Fish gelatin (6.67%, *w*/*v*), glucose (1.6%, *w*/*v*), and β-cyclodextrin (1.6%, *w*/*v*) solutions were prepared separately. Fish gelatin solutions were mixed with glucose or β-cyclodextrin solutions to achieve final concentrations of 5% (*w*/*v*) fish gelatin and 0.4% (*w*/*v*) glucose or β-cyclodextrin. The pH of each mixture was adjusted to 10.0 by the gradual addition of 1.0 mol/L NaOH. The mixtures were then reacted at 50 °C for 2 h under constant magnetic stirring. After the reaction, the samples were diluted with deionized water to a fish gelatin concentration of 4% (*w*/*v*) and cooled to room temperature in an ice-water bath to obtain glycosylated fish gelatin. Equal volumes (1:1, *v*/*v*) of the glycosylated fish gelatin and soybean oil were mixed and homogenized using an IKA high-speed shear homogenizer at 10,000 rpm for 5 min. The resulting emulsions, containing 50% (*v*/*v*) oil and 2% (*w*/*v*) protein, were incubated at 4 °C for 16–18 h to form emulsion gels.

Preparation of surimi gel: The surimi was thawed at 4 °C and coarsely chopped prior to processing. All subsequent blending steps were performed in a vacuum chopping bowl (UMC 5, Stephan Machinery Co., Hamelin, Germany) while maintaining the temperature below 10 °C. The surimi was initially blended without additives for 2 min. Salt (2.5%, *w*/*w*) was then added and blended for another 2 min. FG-Glu or FG-βCD emulsion gel was incorporated at 5%, 10%, or 15% (*w*/*w*). Ice water was introduced to adjust the total moisture content to 78% (*w*/*w*), and the mixture was blended for a final 6 min to achieve a homogeneous surimi paste.

The resulting mixture was subsequently stuffed into 25 mm diameter plastic casings, sealed at both ends, and incubated in a thermostatic water bath (DK-S22, Jinghong Experimental Equipment Co., Ltd., Shanghai, China) at 30 °C for 60 min to form pre-gelled samples. These pre-gels were then immediately heated to 90 °C for 30 min and subsequently cooled in an ice-water mixture to obtain the final gel samples. Surimi gel without added emulsion was used as the control group.

### 2.3. Determination of Color

A colorimeter (CR-400, Konica Minolta Corporation, Japan) was calibrated with a standard white board prior to measuring the color of the surimi gel samples. The parameters recorded included lightness ($L^{*}$), red-green deviation ($a^{*}$), and yellow-blue deviation ($b^{*}$). The whiteness value was calculated according to Equation (1):

(1) $$W\;=\;100\;-\;\sqrt{(100\;-\;L^{*}{)}^{2}\;+\;(a^{*}{)}^{2}\;+\;(b^{*}{)}^{2}}$$

### 2.4. Determination of Gel Strength

The surimi gel samples were allowed to equilibrate at room temperature before being cut into pieces with heights and diameters of 25 mm. Gel strength was evaluated using a texture analyzer (TA.XT Plus Texture Analyzer, Stable Micro Systems, Godalming, UK) with P/0.5 probe, with the following parameters: a pre-test speed of 1 mm/s, a test speed of 1 mm/s, a post-test speed of 10 mm/s; each sample was performed in triplicate.

### 2.5. Determination of Textural Profile Analysis (TPA)

The surimi gel samples were equilibrated at room temperature and cut into pieces of heights and diameters of 25 mm. TPA was conducted using a texture analyzer (TA.XT Plus Texture Analyzer, Stable Micro Systems, UK) with a P/50 probe to evaluate hardness, springiness, cohesiveness, gumminess, and resilience. The parameters used for testing included a pre-test speed of 1 mm/s, a test speed of 5 mm/s, a post-test speed of 1 mm/s, and a compression strain of 30%.

### 2.6. Determination of Juice Loss

Fluid loss comprises cooking loss and pressing loss. The cooking loss was determined according to the method proposed by Zhu [9] with minor modifications. It was calculated using Equation (2) based on the mass difference between the surimi before and after cooking.

(2) $$\mathrm{Cooking}\;\mathrm{loss}\;(\%)=\frac{\mathrm{Pre-cooking}\;\mathrm{weight}-\mathrm{Mass}\;\mathrm{after}\;\mathrm{cooking}}{\mathrm{Pre-cooking}\;\mathrm{weight}}\;\times \;100$$

Gel samples were sliced into approximately 2 mm-thick sections and weighed (recorded as *W*_1_). Each slice was placed between two layers of filter paper (one on the top and one on the bottom). A texture analyzer fitted with a P/50 probe was used to compress the sample with a constant force of 2 kg for 20 s. After compression, the gel slice was re-weighed (*W*_2_). The pressing loss was calculated using Equation (3).

(3) $$\mathrm{crushing}\;\mathrm{loss}\;\mathrm{rate}\;(\%)=\frac{W_{1}-W_{2}}{W_{1}}\;\times \;100\%$$

### 2.7. Determination of Chemical Forces

The chemical forces in surmi gels were measured following the method described by Du [10] with minor modifications: 1 g sample was combined with 10 mL of extraction solutions: S_1_ (0.05 M NaCl), S_2_ (0.6 M NaCl), S_3_ (0.6 M NaCl + 1.5 M urea), and S_4_ (0.6 M NaCl + 8 M urea). Each extraction solution was added to the sample, which was then homogenized in an ice bath at 2800 rpm for 0.3 min. The mixture was subsequently centrifuged at 10,000 rpm for 15 min at 4 °C to obtain the supernatant. The protein concentration in the supernatant was determined using a BCA kit. The content of each type of chemical interaction was calculated using Equation (4).

(4) $$\left\{\begin{array}{c} Nonspecific\;association=c\left(S_{1}\right)\\ Ionic\;bond=c\left(S_{2}\right)-c\left(S_{1}\right)\\ Hydrogen\;bond=c\left(S_{3}\right)-c\left(S_{2}\right)\\ Hydrophobic\;interaction=c\left(S_{4}\right)-c\left(S_{3}\right) \end{array}\right.$$

where c represents the protein concentration.

### 2.8. Determination of Protein Secondary Structure

Following the method described by Mi [11] with slight modifications, surimi gel samples were freeze-dried and pulverized. Approximately 2 mg of sample powder was thoroughly mixed with KBr at a 1:50 (*w*/*w*) ratio and pressed into transparent pellets. Protein conformation was examined using a Fourier transform infrared spectrometer (FT/IR-4700, JASCO, Tokyo, Japan). Spectra were collected in the range of 400–4000 cm^−1^ with a resolution of 4 cm^−1^ after 32 scans. The amide I region (1600–1700 cm^−1^) was subsequently analyzed using PeakFit software (version 4.12) to quantify the relative proportions of protein secondary structures.

### 2.9. Determination of Moisture Distribution

A 10 g surimi gel was placed in a 40 mm NMR tube, and the transverse relaxation time (T_2_) of the internal water within the gel was measured using a low-field nuclear magnetic resonance (NMR) analyzer. The experimental parameters were established as follows: number of scans—2; magnet temperature—32 °C; resonance frequency—23.4 MHz; magnetic field strength—0.55 T; repetition time—10,000 ms; echo time—0.2 ms [12].

### 2.10. Scanning Electron Microscope Observation

The microstructure of surimi gel was observed using the following procedure of Mi et al. [13]: Gel samples were trimmed into 1 mm^3^ cubes and fixed in 2.5% glutaraldehyde at 4 °C for 24 h. After fixation, the samples were washed three times (15 min each) with 0.1 M phosphate buffer (pH 7.2). Dehydration was then carried out using a graded ethanol series (30%, 50%, 70%, 80%, 90%, and absolute ethanol), followed by a tert-butanol series (ethanol–tert-butanol mixtures at 3:1, 1:1, and 1:3, and finally pure tert-butanol). The dehydrated sample was coated with a thin layer of pure tert-butanol, rapidly frozen at −80 °C, and lyophilized. Prior to imaging, the dried sample was sputter-coated with gold under vacuum and observed using a scanning electron microscope (S-3400N, Hitachi, Tokyo, Japan) at an acceleration voltage of 10 kV and a magnification of 8000×.

### 2.11. Data Processing

All experiments were performed in replicates, and the results are expressed as the mean ± standard deviation. Data were statistically analyzed using SPSS software (Version 26.0). One-way analysis of variance was used to evaluate significant differences among groups, followed by Duncan’s multiple range test for post hoc comparisons at a statistical significance level of *p* < 0.05. Figures were prepared using Origin 2023 software.

## 3. Results and Discussion

### 3.1. Color Change

Whiteness (*W*) is a crucial sensory attribute that determines the quality of surimi products, and consumers generally favor products that exhibit higher whiteness values [14]. Both lightness (L*) and whiteness (*W*) reflect internal biochemical changes in surimi gel, thereby influencing the consumer sensory evaluation.

As shown in Table 1, compared to the control group, both FG-Glu and FG-βCD emulsion gels significantly enhanced the L*, a*, b* and whiteness *W* values of surimi gels, indicating two emulsion gel systems could significantly change the appearance value of surimi gel. As the addition level of the emulsion gels increased, both *W* and lightness L* values exhibited a significant increase (*p* < 0.05), with FG-βCD demonstrating a more pronounced enhancement in the *W* value. These results suggest that incorporating glycated FG emulsion gels contributes to changing the *W* values of surimi gels, thereby improving their visual appeal.

The whiteness of surimi gels is influenced by the type and concentration of exogenous additives, as well as the optical properties of the gel surface [15]. In the present study, the observed increase in whiteness (*W*) may be partly explained by the inherent milky-white color of the FG-Glu and FG-βCD emulsion gels themselves. An increase in the concentration of emulsified vegetable oil has also been associated with enhanced gel whiteness [16]. Moreover, the uniform dispersion of FG-Glu and FG-βCD emulsion droplets within the protein matrix likely contributes to stronger light scattering at the gel surface, further improving its whiteness.

### 3.2. Analysis of Gel Strength and Texture Properties

Textural properties are key indicators for evaluating surimi products and directly influence consumer preference and acceptance [17]. As shown in Table 2, the gel strength and texture profile analysis (TPA) parameters of surimi gels are normally enhanced by the addition of FG-Glu and FG-βCD emulsion gels, which were significantly higher than those of the control group. For the FG-Glu group, the gel and TPA parameters were first increased with the FG-Glu emulsion contents from 5% to 10%, and then decreased. At a 10% addition level gave surimi gels the highest gelling properties; both emulsion gels significantly improved the gel strength of surimi gels. For the FG-βCD group, the gel strength and TPA parameters were increased with the FG-βCD emulsion gels, and 15% gave the surimi gels the highest values. This might be due to the addition of glycated fish gelatin emulsion, which was filled within the protein gel network during heating [18]. The presence of oil droplets may promote the exposure of hydrophobic protein groups, enhancing hydrophobic interactions between proteins and thus increasing the strength of the surimi gels. Similarly, Min Chen et al. [19] reported that whey protein isolate and safflower seed oil emulsions also significantly increased the gel strength of surimi gels. Pei et al. [20] also demonstrated that the Tilapia myofibrillar protein-stabilized emulsion gels could similarly reinforce lipid-enhanced surimi.

However, at the highest emulsion gel level (15%), two emulsion gel systems showed different tendencies. For the FG-Glu, the gel strength began to decline, indicating that the binding capacity of fish proteins approached saturation. Excessive oil droplets may interfere with protein cross-linking, resulting in reduced gel strength [16]. Xu et al. [19] found that when sodium caseinate emulsion content exceeded 5%, it negatively impacted the gel properties of surimi. Gani et al. [21]. noted that the hardness and gel strength of surimi gels are closely related to protein concentration. When the moisture content remains constant, the addition of oil decreases protein concentration, leading to diminished hardness.

In contrast, for the FG-βCD, 15% emulsion gel positively influenced surimi gel formation. This divergence underscores the comparative advantage of the cyclic β-CD modifier over linear glucose. The unique ring cavity and abundant hydroxyl groups of β-CD likely facilitate superior hydrogen bonding and spatial stability, allowing the emulsion droplets to function as active reinforcements within the myofibrillar protein matrix rather than inducing structural interference. This structural synergy resulted in a significantly more cohesive and resilient network, as validated by the dense and uniform microstructures observed at high concentrations.

Furthermore, FG-βCD treatment results in a more substantial increase in gel hardness relative to FG-Glu. This difference can be attributed to the smaller emulsion droplet size observed in the FG-βCD system, as noted in our earlier study. Smaller oil droplets disperse more uniformly throughout the protein gel matrix, reducing the interference with protein–protein interactions during network formation [22]. Consequently, adding an optimal amount of emulsion—particularly from the FG-βCD group—can simultaneously improve both the gel strength and textural profile of surimi gels.

### 3.3. Changes in Juice Loss

**Figure 1:**
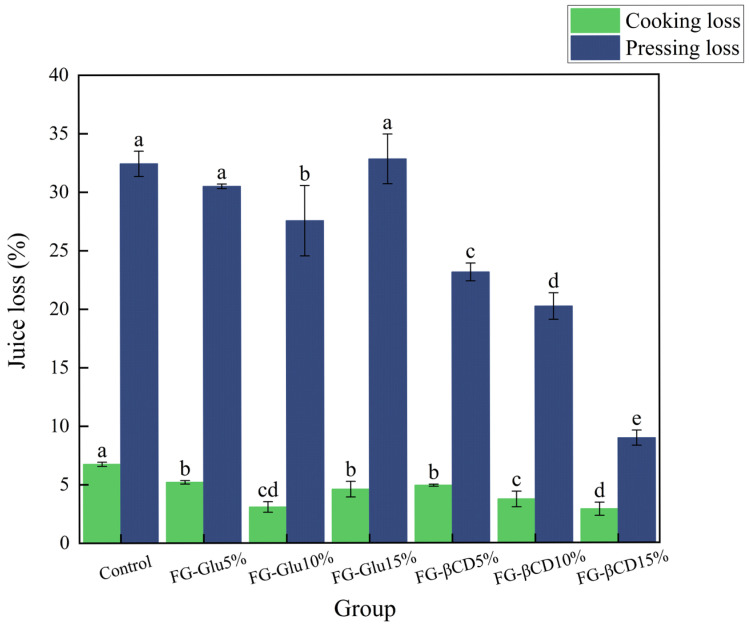
Effect of glycosylation of FG emulsion gel additions on the loss of juice from surimi gels. Note: Significance analysis was performed as a between-group analysis, where different lowercase letters indicate statistically significant differences (*p* < 0.05).

The fluid loss of surimi gels consists of cooking loss and pressing loss, where cooking loss refers to the reduction in water, protein, and other components during the heating process of surimi gels. The cooking loss refers to the reduction in water, protein, and other components during the heating process of surimi gels [23]. Variations in fluid loss directly affect the gel properties and sensory quality of the product [24]. As shown in Figure 1, all samples (except the 15% FG-Glu group) exhibited significantly lower fluid loss compared to the control group (*p* < 0.05), indicating that the glycosylated FG emulsion gels played a positive role in reducing fluid loss in surimi gels.

For the FG-Glu group, fluid loss showed a significant decrease in the addition level, which had increased from 5% to 10%, indicating that a moderate amount of FG-Glu emulsion enhances the water- and fat-retention capacity of surimi gels. When the addition level was 15%, the pressing loss was increased noticeably, whereas cooking loss remained largely unchanged. The increase in pressing loss observed in the 15% FG-Glu group further corroborates the chemical force findings. The decline in hydrogen bonds below control levels suggests a weakened ability of the protein scaffold to bind water molecules effectively. This structural vulnerability, likely caused by the interference of excessive emulsion droplets at high concentrations, leads to reduced fluid retention under mechanical pressure, whereas the FG-βCD system provides a more stable environment for water entrapment due to its sustained molecular interactions.

In contrast, the FG-βCD group exhibited a consistent reduction in both cooking and pressing losses with the increased level of FG-βCD emulsion gel, showing higher stability than those of the FG-Glu group and control. This superior performance is intrinsically associated with the structural attributes of β-CD, which promote a conformational shift toward more ordered protein secondary structures, characterized by significantly increased α-helix and β-sheet contents. Unlike the linear glucose system, which exhibits a functional threshold at 10%, the β-CD-modified gelatin emulsion effectively entraps water molecules within a more compact three-dimensional protein scaffold. These findings highlight the potential of cyclic glycosylated carriers as high-efficiency functional additives for optimizing the texture and stability of aquatic food products.

As the levels of FG-Glu and FG-βCD incorporation increased, the cooking loss of surimi gels decreased significantly, a trend consistent with the findings of Zhao et al. [25]. Emulsions are known to effectively enhance the water- and fat-binding capacity of myofibrillar protein gels. This reduction in cooking loss may also be linked to the formation of a more compact gel structure [26], which, as noted by Gao et al. [27], generally exhibits improved water retention. Notably, at the 15% addition level, the FG-Glu group showed increased fluid loss, whereas the FG-βCD group demonstrated the opposite behavior. This divergence may be attributed to the structural properties of β-cyclodextrin, which facilitate the binding of both fats and water. The presence of abundant hydroxyl groups in β-cyclodextrin promotes interactions with water molecules, thereby enhancing the water-holding capacity of the gel network.

### 3.4. Analysis of Chemical Forces

The chemical interactions in surimi gels can be characterized by soluble protein content, including non-specific associations, ionic bonds, hydrogen bonds, and hydrophobic interactions [28]. As shown in Table 3, the addition of emulsion gels could increase the non-specific associations, hydrogen bond and hydrophobic interactions of surimi gels.

The non-specific associations and hydrophobic interactions were predominant in all samples, highlighting their significant roles in the formation of the surimi gel network. Zhu et al. [29] also reported similar findings, where the incorporated emulsions containing perilla and soybean oil emulsions incorporated into myofibrillar protein gels also exhibited significantly increased levels of non-specific associations and hydrophobic interactions.

When the added FG-Glu and FG-βCD contents were increased from 5% to 10%, both nonspecific associations and hydrogen bonding within the surimi gels increased significantly (*p* < 0.05). Hydrophobic interactions also increased in both groups, though this effect was more pronounced in gels containing FG-βCD. At 15% addition level, the FG-βCD group maintained higher nonspecific associations and hydrogen bonding, while its hydrophobic interactions declined relative to the 10% level. In contrast, the FG-Glu group exhibited a significant reduction in both nonspecific associations and hydrogen bonding at the 15% level. Notably, the hydrogen bond content in this group decreased to 0.10 mg/mL, which was lower than that of the control group (0.13 mg/mL). This suggests that high concentrations of glucose-modified gelatin emulsion may interfere with the established protein–protein interactions, potentially hindering the formation of a stable three-dimensional network and reducing the overall water-holding capacity of the surimi gel. Conversely, the FG-βCD group maintained a robust increase in hydrogen bonding (reaching 0.24 mg/mL), reinforcing the superiority of cyclodextrin-modified systems in stabilizing the gel matrix through enhanced molecular cross-linking.

In this study, nonspecific associations are defined as the protein fraction represented by the stable residues remaining after the sequential extraction of ionic, hydrogen, and hydrophobic bonds using the S1–S4 buffer systems. These associations, alongside hydrophobic interactions, serve as the predominant chemical forces that reinforce the three-dimensional scaffold and ensure the structural integrity of the surimi gel network [13].

Heating is the primary driver that induces conformational changes in proteins, leading to the exposure of internal hydrophobic groups, especially in myosin. These exposed groups subsequently aggregate via hydrophobic interactions to construct the gel network [30]. These hydrophobic groups could be aggregated through the hydrophobic interactions, contributing to gel network formation. The increase in hydrophobic interactions with various concentrations of FG-Glu and FG-βCD suggests that emulsion gels can promote protein unfolding in surimi. Jiao et al. [31] found that fish oil altered protein conformation in silver carp surimi by promoting the exposure of hydrophobic groups in protein molecules.

The observed increase in hydrogen bonds may be attributed to conformational and structural changes induced by the addition of FG-Glu and FG-βCD, thereby enhancing the number of hydrogen bonds [32]. Although hydrogen bonds are relatively weak dipole interactions, they are essential for stabilizing the bound water in the system [33]. Zhao et al. [34] also reported that the incorporation of emulsions promoted the formation of hydrophobic interactions and rearrangement of hydrogen bonds, consequently improving the strength of surimi gels.

Ionic bonds arise from electrostatic attractions between positively and negatively charged amino acid residues in proteins. The observed decline in ionic bond content with increasing emulsion gel addition suggests that the incorporated gels interfere with protein cross-linking, likely by masking charged groups or altering the local electrostatic environment [35]. Overall, the surimi gels exhibited strengthened non-specific associations and hydrogen bonding—most notably at 10% FG-Glu and 15% FG-βCD content—whereas hydrophobic interactions reached their maximum at the 10% addition level for both groups, while ionic bonding was concurrently reduced.

### 3.5. Changes in Protein Secondary Structure

Protein secondary structure, which describes the local folding of the polypeptide backbone, plays a key role in determining protein functionality [36]. In Fourier-transform infrared (FTIR) spectroscopy, the Amide I band (1600–1700 cm^−1^) is widely used to characterize secondary structure components: α-helices (1650–1660 cm^−1^), β-sheets (1600–1640 cm^−1^), β-turns (1660–1700 cm^−1^), and random coils (1640–1650 cm^−1^) [37]. In this study, Gaussian curve fitting was applied to the Amide I region to quantify the relative proportions of these structures in the surimi gels.

**Figure 2:**
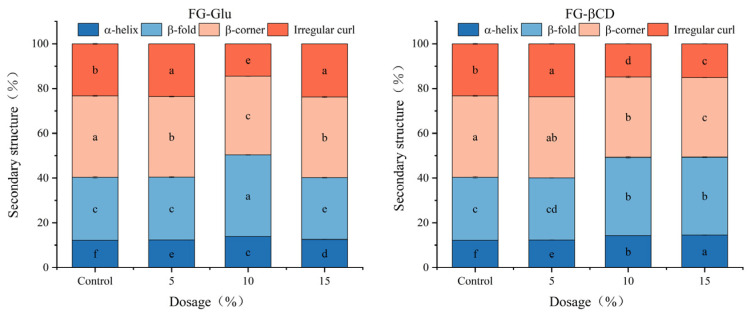
Effect of FG-Glu and FG-βCD emulsion gel addition on the secondary structure of surimi. Note: Significance analysis was performed as a between-group analysis, where different lowercase letters indicate statistically significant differences (*p* < 0.05).

As shown in Figure 2, β-sheets and β-turns are the primary secondary structures in fish paste gels, contributing to the formation of a dense protein network [35]. Compared to the control group, adding FG-Glu and FG-βCD emulsion gels significantly increased the proportion of α-helix and β-sheet (*p* < 0.05) while decreasing the content of β-turns and random coil (*p* < 0.05). These changes indicate a shift from disordered structures (β-turns and random coils) toward more ordered conformations (α-helices and β-sheets), likely due to enhanced protein–protein interactions and hydrogen bonding during gelation. Furthermore, emulsion gels with higher addition levels typically further amplify these structural effects.

In the FG-Glu group, the ratio of α-helix to β-sheet increased initially and then decreased with increasing emulsion content, peaking at 10% addition (13.84% and 36.49%, respectively). Concurrently, the β-turn content exhibited a slight downward trend, while the random coil content initially decreased and then increased, reaching its lowest level (14.43%) at the 10% addition rate.

A different trend was observed in the FG-βCD group. The α-helix content steadily increased with rising addition levels, peaking at 14.51% at a 15% addition rate. The β-sheet content initially increased and stabilized, reaching a maximum of 35.00% at a 10% addition rate. β-turn content gradually decreased, while the change pattern of random coils resembled that of the FG-Glu group—first decreasing then increasing, with its minimum value (14.79%) also occurring at the 10% addition level. These phenomena are likely attributed to the stabilizing environment provided by the FG-Glu or FG-βCD emulsion gels, which facilitates the reorganization of surimi proteins into more ordered secondary structures. While α-helices are primarily stabilized by intramolecular hydrogen bonds, the incorporation of these glycosylated emulsion gels promotes the formation of a dense protein network that supports such conformational stability.

In addition, both native and partially unfolded α-helices, as well as β-sheets that develop during heat treatment, are largely stabilized through hydrogen bonding [38]. Consistent with this structural shift, Cao [39] observed that a higher β-sheet content corresponds to better gelation performance. This trend was further supported by Mi et al. [11], who found that starch-based emulsions with varying amylose content similarly shift protein secondary structures toward more ordered forms to enhance surimi gel stability. These comparative results indicate that glycosylated FG emulsion gels function effectively as structural modifiers, promoting the transition of random coils into stable α-helix and β-sheet structures. During heating, intensified protein–lipid and protein–protein interactions facilitate β-sheet formation through intra- and intermolecular interactions [40]. The presence of lipids exposes more hydrophobic side chains of surimi proteins, which associate with oil droplets, thereby altering protein conformation [16].

In terms of structural dynamics, β-turns and random coils reflect greater protein flexibility and a less compact architecture [41]. The control group, which contained higher levels of these disordered components, exhibited correspondingly lower structural stability and a less cohesive gel network relative to the emulsion-gel-fortified samples. Together, these structural shifts indicate that FG-Glu and FG-βCD emulsions stabilize protein conformation, promote intermolecular cross-linking, and ultimately enhance the overall gel strength of surimi gels.

### 3.6. Changes in Moisture Distribution

**Figure 3:**
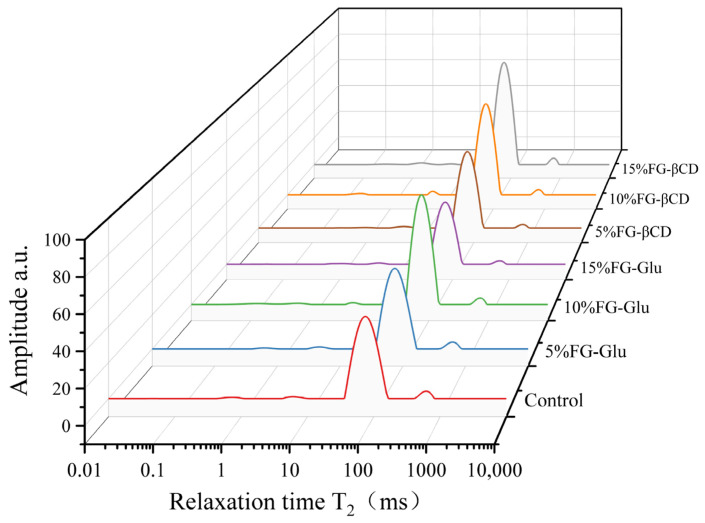
Effect of glycosylation FG emulsion gel addition on T2 relaxation time of surimi gels.

Transverse relaxation time (T2), as determined by low-field nuclear magnetic resonance (LF-NMR), characterizes the distribution, mobility, and exchange of water in surimi gels based on the relaxation behavior of hydrogen protons. The influence of FG-Glu and FG-βCD emulsion gels on the relaxation signal intensity of the gels is presented in Figure 3.

Compared with the control group, incorporation of glycosylated fish gelatin emulsion gels (FG-glu and FG-βCD) at levels of 5–15% resulted in a shift of the main T_2_ relaxation peak towards shorter relaxation times (Figure 3). Concurrently, the proportion of less mobile water (P_22_) increased significantly, while the proportion of free water (P_23_) correspondingly decreased, with little change in bound water (P_21_) (Table 4). These findings indicate enhanced binding of water molecules within the gel matrix, reflecting the formation of a more compact and uniform three-dimensional network structure with superior water retention capacity. This improvement may be attributed to strengthened intermolecular interactions between glycosylated FG emulsion droplets and myofibrillins, including hydrogen bonds, hydrophobic associations, and disulfide bonds, which facilitate the construction of denser gel scaffolds capable of effectively trapping water. Wu et al.’s research also indicates that emulsion gels form denser networks, increasing capillary forces and enhancing water retention [42].

Significant differences exist between the two glycosylated emulsions. For FG-Glu, P_22_ reached its maximum (96.28%) at 10% addition, then decreased slightly at 15% addition, while P_23_ was lowest (1.50%) at 10% addition. In FG-βCD, P_22_ content increased in a dose-dependent manner, reaching 96.30% at 15% addition with the lowest free water content (1.44%). This discrepancy likely stems from differing structural characteristics of the glycosylating agents: linear glucose modifications achieve optimal interfacial compatibility at moderate levels but induce slight overaggregation at high doses, whereas β-cyclodextrin’s ring cavity possesses superior spatial stability, maintaining protein-emulsion interactions even at high concentrations. Consequently, 10% FG-glu demonstrated the most pronounced enhancement of water-holding capacity in surimi gels, while 15% FG-βCD exhibited superior enhancement with stronger dose dependency.

### 3.7. Microstructure Analysis

**Figure 4:**
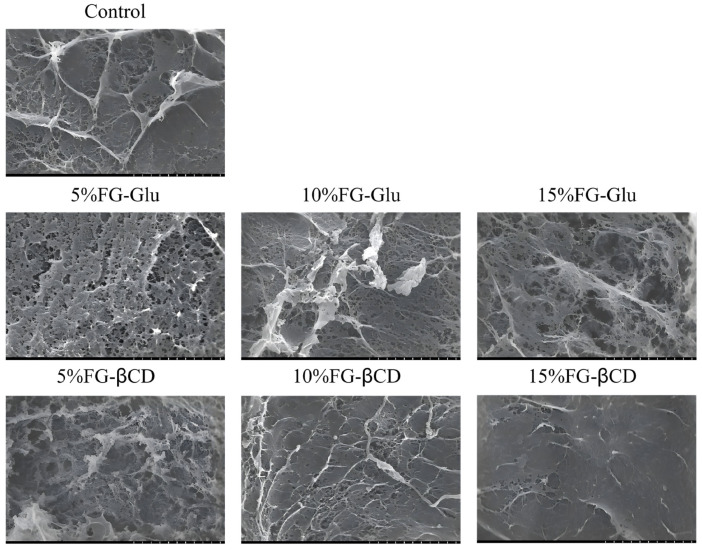
Effect of glycosylation FG emulsion gel addition on the microstructure of surimi gels.

Microstructural analysis (Figure 4) revealed that the inclusion of FG-Glu and FG-βCD emulsion gels substantially altered the architecture of the surimi gel network. As the level of FG-βCD increased, the gel matrix grew progressively denser, with smaller pores and a smoother, more uniform surface. These structural refinements align closely with the observed gains in gel strength and texture profile analysis (TPA) parameters, supporting the established view that a more compact network generally leads to stronger gels [11]. Relative to the untreated control, surimi gels with 10% FG-Glu displayed a dense and homogeneous network, featuring clustered aggregates and maintained structural integrity. This improvement can be attributed to emulsion droplets occupying voids within the protein matrix, which reinforces the three-dimensional network [20]. The formation of this well-organized structure was further supported by intensified nonspecific associations, hydrogen bonding, and hydrophobic interactions.

However, at 15% FG-Glu addition, the gel microstructure was disrupted, with larger pores and a more fragile network evident. This deterioration is presumably caused by excessive emulsion content interfering with protein cross-linking and proper network formation [16]. Collectively, these findings suggest that incorporating emulsion gels at appropriate levels promotes the formation of a dense and well-organized gel matrix in surimi, which contributes significantly to its desired textural properties.

### 3.8. Correlation Analysis Between Data Sets

**Figure 5:**
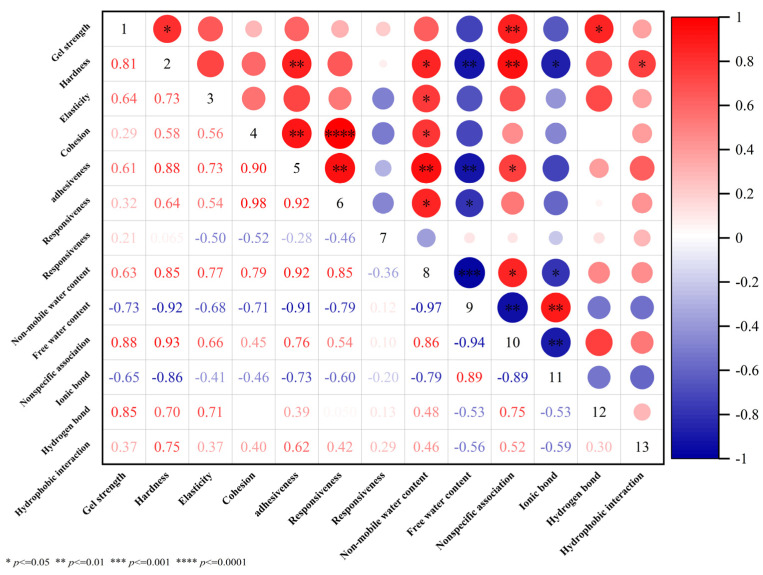
Correlation Analysis of Surimi Gel Data Sets with Added Glycosylated FG Emulsion Gel.

The correlation heatmap (Figure 5) illustrates the relationships among various physicochemical properties of hairtail surimi gels, offering statistical support for the principal findings of this study. Gel strength showed a significant positive correlation (*p* < 0.05) with key textural parameters—including hardness, adhesiveness, springiness, cohesiveness, and resilience—indicating that enhanced gel strength corresponds to an overall improvement in texture. Furthermore, the gel quality exhibited a strong positive correlation with immobilized water content and a corresponding negative correlation with free water content. These correlations align with the proposed mechanism in which emulsion gels improve gel performance by restricting water mobility and enhancing water retention within the network.

At the molecular interaction level, nonspecific associations, hydrogen bonds, and hydrophobic interactions all showed strong positive correlations with both gel strength and key textural attributes. These same interactions were positively linked to immobilized water content and inversely related to free water content, reinforcing their critical contribution to forming and stabilizing a dense gel network. In contrast, ionic bonds displayed a significant negative correlation with gel strength, texture, and immobilized water, implying that the addition of emulsion gels modifies protein cross-linking patterns, reduces reliance on ionic bonds, and allows other stabilizing forces to become predominant. In summary, the correlation analysis clarifies how glycosylated fish gelatin emulsion gels refine the internal chemical interaction profile of surimi gels and promote more effective water binding within the protein matrix. These coordinated effects collectively contribute to greater gel strength, enhanced textural properties, and reduced fluid loss, raising the overall quality of the final product.

The correlation heatmap provides robust statistical evidence for the intrinsic mechanisms governing hairtail surimi gel quality. Gel strength and key textural parameters exhibited significant positive correlations (*p* < 0.05) with the content of α-helices and β-sheets, suggesting that the transition toward more ordered protein conformations is a prerequisite for textural enhancement. Furthermore, the strong positive correlation between non-mobile water (P22) and both nonspecific associations and hydrophobic interactions elucidates the moisture-retention mechanism; these intensified chemical forces facilitate the construction of a dense three-dimensional scaffold that effectively traps water molecules, resulting in the observed 69.81% reduction in juice loss. In contrast, the significant negative correlation between ionic bonds and gel strength indicates a strategic shift in the molecular stabilization energy of the surimi matrix. This statistical profile confirms that glycosylated fish gelatin emulsion gels—particularly the FG-βCD system—optimize gel performance by promoting favorable protein–lipid–water interactions and reinforcing the network through more robust covalent-like and hydrophobic cross-linking.

## 4. Conclusions

This study investigated the effects of emulsifying gel at different addition levels (5%, 10%, 15%) on the characteristics of hairtail fish paste. The results showed that the proper contents of FG-Glu and FG-βCD emulsifying gels could significantly improve the gel strength, texture, color, and water retention of surimi gels. FG-βCD demonstrated overall superiority over FG-Glu in texture improvement and water retention. The Secondary structure analysis indicated that increased emulsion gels (from 5% to 10%) could increase the α-helix and β-sheet content of surimi protein, while reducing random coils. Hydrophobic interactions and non-specific binding dominated gel formation. With increasing addition levels of emulsion gels, the hydrophobic interactions, non-specific binding, and hydrogen bonding increased, while ionic bonding decreased. The emulsified gel promoted gel network formation and stability by regulating intermolecular forces.

## Figures and Tables

**Table 1:** Effect of the addition of glycosylated FG emulsion gels on the color of surimi gels.

Sample	Dosage	$L^{*}$	$a^{*}$	$b^{*}$	W
Control Group	0	54.94 ± 1.88 ^e^	−0.94 ± 0.05 ^d^	7.61 ± 0.29 ^c^	54.29 ± 1.80 ^e^
FG-Glu	5%	61.36 ± 2.12 ^d^	−0.71 ± 0.00 ^c^	8.88 ± 0.55 ^b^	60.34 ± 1.95 ^d^
	10%	67.97 ± 1.24 ^c^	−0.53 ± 0.05 ^a^	9.20 ± 0.54 ^b^	66.66 ± 1.05 ^c^
	15%	74.36 ± 0.75 ^ab^	−0.61 ± 0.02 ^b^	10.04 ± 0.34 ^a^	72.46 ± 0.58 ^a^
FG-βCD	5%	62.91 ± 0.43 ^d^	−0.51 ± 0.03 ^a^	8.06 ± 0.23 ^c^	62.04 ± 0.38 ^d^
	10%	72.41 ± 0.22 ^b^	−0.61 ± 0.04 ^b^	10.17 ± 0.28 ^a^	70.59 ± 0.11 ^b^
	15%	75.16 ± 0.29 ^a^	−0.65 ± 0.01 ^b^	10.70 ± 0.08 ^a^	73.00 ± 0.29 ^a^

Note: Significance analysis was performed as a between-group analysis, where different lowercase letters indicate statistically significant differences (*p* < 0.05).

**Table 2:** Effect of glycosylation FG emulsion gel addition on gel strength and TPA of surimi gels.

Sample	Dosage	Gel Strength (N)	Hardness (N)	Elasticity (%)	Cohesion (%)	Adhesiveness (N)	Resilience
Control Group	0	9.02 ± 0.38 ^e^	4.57 ± 0.31 ^d^	0.88 ± 0.05 ^ab^	0.45 ± 0.01 ^bc^	2.05 ± 0.14 ^c^	0.18 ± 0.01 ^bc^
FG-Glu	5%	11.67 ± 0.21 ^d^	5.20 ± 0.10 ^c^	0.85 ± 0.05 ^b^	0.50 ± 0.14 ^abc^	2.63 ± 0.80 ^bc^	0.23 ± 0.08 ^abc^
	10%	13.48 ± 0.27 ^b^	6.01 ± 0.01 ^a^	0.94 ± 0.02 ^a^	0.55 ± 0.01 ^ab^	3.32 ± 0.05 ^ab^	0.26 ± 0.01 ^ab^
	15%	8.90 ± 0.42 ^e^	5.48 ± 0.09 ^b^	0.89 ± 0.02 ^ab^	0.58 ± 0.08 ^a^	3.18 ± 0.39 ^ab^	0.29 ± 0.05 ^a^
FG-βCD	5%	11.28 ± 0.47 ^d^	5.31 ± 0.05 ^bc^	0.88 ± 0.05 ^ab^	0.41 ± 0.05 ^c^	2.20 ± 0.29 ^c^	0.18 ± 0.02 ^c^
	10%	12.68 ± 0.68 ^c^	6.17 ± 0.02 ^a^	0.90 ± 0.04 ^ab^	0.50 ± 0.01 ^abc^	3.10 ± 0.03 ^ab^	0.23 ± 0.01 ^abc^
	15%	14.33 ± 0.33 ^a^	6.20 ± 0.21 ^a^	0.94 ± 0.01 ^a^	0.57 ± 0.03 ^ab^	3.53 ± 0.30 ^a^	0.28 ± 0.01 ^a^

Note: Significance analysis was performed as a between-group analysis, where different lowercase letters indicate statistically significant differences (*p* < 0.05).

**Table 3:** Effect of glycosylation FG emulsion gel addition on the chemical force of surimi gels.

Sample	Dosage (*w*/*w*)	Nonspecific Association (mg/mL)	Ionic Bond (mg/mL)	Hydrogen Bond (mg/mL)	Hydrophobic Interaction (mg/mL)
Control Group	0	1.09 ± 0.01 ^e^	0.63 ± 0.05 ^a^	0.13 ± 0.02 ^c^	1.06 ± 0.02 ^e^
FG-Glu	5%	1.33 ± 0.05 ^d^	0.27 ± 0.02 ^b^	0.12 ± 0.02 ^c^	1.12 ± 0.010 ^d^
	10%	1.51 ± 0.02 ^a^	0.23 ± 0.01 ^c^	0.20 ± 0.03 ^b^	1.24 ± 0.03 ^b^
	15%	1.31 ± 0.03 ^d^	0.20 ± 0.01 ^d^	0.10 ± 0.02 ^c^	1.24 ± 0.02 ^b^
FG-βCD	5%	1.39 ± 0.05 ^c^	0.20 ± 0.02 ^d^	0.20 ± 0.02 ^b^	1.11 ± 0.02 ^d^
	10%	1.44 ± 0.01 ^b^	0.13 ± 0.01 ^e^	0.20 ± 0.01 ^b^	1.45 ± 0.01 ^a^
	15%	1.52 ± 0.00 ^a^	0.08 ± 0.02 ^f^	0.24 ± 0.01 ^a^	1.19 ± 0.01 ^c^

Note: Significance analysis was performed as a between-group analysis, where different lowercase letters indicate statistically significant differences (*p* < 0.05).

**Table 4:** Effect of glycosylated FG emulsion gel addition on the relative moisture content of different states of surimi gels.

Sample	Dosage	P_21_ (%)	P_22_ (%)	P_23_ (%)
Control Group	0	2.27 ± 0.05 ^bc^	93.77 ± 0.02 ^e^	3.97 ± 0.06 ^a^
FG-Glu	5%	2.63 ± 0.29 ^a^	94.77 ± 0.35 ^d^	2.55 ± 0.11 ^b^
	10%	2.22 ± 0.16 ^bc^	96.28 ± 0.19 ^a^	1.50 ± 0.04 ^d^
	15%	2.15 ± 0.13 ^c^	95.80 ± 0.08 ^b^	2.05 ± 0.17 ^c^
FG-βCD	5%	2.53 ± 0.20 ^ab^	94.89 ± 0.10 ^d^	2.58 ± 0.11 ^b^
	10%	2.71 ± 0.15 ^a^	95.22 ± 0.18 ^c^	2.07 ± 0.12 ^c^
	15%	2.26 ± 0.04 ^bc^	96.30 ± 0.16 ^a^	1.44 ± 0.12 ^d^

Note: P21 represents the bound water content; P22 represents the less mobile water content; P23 represents the free water content. Significance analysis was performed as a between-group analysis, where different lowercase letters indicate statistically significant differences (*p* < 0.05).

## Data Availability

The original contributions presented in the study are included in the article; further inquiries can be directed to the corresponding author.
